# Increased excitability and altered action potential waveform in cerebellar granule neurons of the Ts65Dn mouse model of Down syndrome

**DOI:** 10.1016/j.brainres.2012.05.027

**Published:** 2012-07-17

**Authors:** Maria M. Usowicz, Claire L.P. Garden

**Affiliations:** School of Physiology & Pharmacology, University of Bristol, University Walk, Bristol, BS8 1TD, UK

**Keywords:** GC, granule cell, DS, Down syndrome, PC, Purkinje cell, AP, action potential, EPSC, excitatory postsynaptic current, P, postnatal day, *R*_in_, input resistance, *C*_in_, input capacitance, Down syndrome, Cerebellum, Granule cell, Hypoplasia

## Abstract

Down syndrome (DS) is characterized by intellectual disability and impaired motor control. Lack of coordinated movement, poor balance, and unclear speech imply dysfunction of the cerebellum, which is known to be reduced in volume in DS. The principal cause of the smaller cerebellum is a diminished number of granule cells (GCs). These neurons form the ‘input layer’ of the cerebellar cortex, where sensorimotor information carried by incoming mossy fibers is transformed before it is conveyed to Purkinje cells and inhibitory interneurons. However, it is not known how processing of this information is affected in the hypogranular cerebellum that characterizes DS. Here we explore the possibility that the electrical properties of the surviving GCs are changed. We find that in the Ts65Dn mouse model of DS, GCs have a higher input resistance at voltages approaching the threshold for firing, which causes them to be more excitable. In addition, they fire narrower and larger amplitude action potentials. These subtly modified electrical properties may result in atypical transfer of information at the input layer of the cerebellum.

## Highlights

► Granule neurons in the cerebellum of the Ts65Dn mouse model of Down syndrome. ► Action potential firing is evoked with smaller current injections. ► Action potentials are narrower and have larger amplitudes.

## 1 Introduction

Down syndrome (DS) describes a collection of disabilities that include mental retardation and motor incoordination. It is due to the inheritance of an additional copy of all or part of chromosome 21 (trisomy 21; OMIM ID: 190685) and occurs in different populations in 1 per 370 to 1700 live births (Cocchi et al., 2010; O'Nuallain et al., 2007; Parker et al., 2010). Impaired motor coordination in DS is evident as limited fine motor control, delays in the acquisition of gross and fine motor skills, dysarthria (the unclear articulation of words), strabismus (squint), nystagmus (oscillating eye movements), and altered balance and gait (Frith and Frith, 1974; Henderson et al., 1981; Spano et al., 1999; references in Galante et al., 2009).

The lack of coordination and poor balance implicate dysfunction of the cerebellum, a key brain structure involved in the control of movement. This inference is supported by the finding that in individuals with DS, the volume of the cerebellum and the density of GCs therein are reduced by one third and one quarter respectively (Aylward et al., 1997; Baxter et al., 2000; Jernigan and Bellugi, 1990; Pinter et al., 2001; Raz et al., 1995). Moreover, modeling of the triplication of genes on human chromosome 21 in DS, by triplication of differing numbers of orthologous genes in mice, generates different mouse models (for example, Ts65Dn, Ts1Cje, Ts1Rhr, Tc1) with varying degrees of decreased cerebellar volume, lower GC density and altered behavior (Dierssen et al., 2009; Galante et al., 2009; Haydar and Reeves, 2011; Lana-Elola et al., 2011; Moldrich et al., 2007). These changes may be accompanied by changes in cerebellar gene expression (Laffaire et al., 2009; Moldrich et al., 2007) and in the number and morphology of Purkinje cells (PCs), the class of cerebellar neuron that integrates input from GCs, as well as other cells, and produces the sole output from the cerebellar cortex (Baxter et al., 2000; Necchi et al., 2008). It is not known if these anatomical and transcriptional modifications are accompanied by alterations in the function of the surviving GCs, which constitute the first stage at which sensorimotor signals transmitted to the cerebellum by mossy fibers (MFs) are processed (Arenz et al., 2009).

We investigated the input–output characteristics of GCs in the young adult Ts65Dn mouse, a model which replicates the deficit of GCs observed in DS and is the most widely studied model of DS (Baxter et al., 2000; Dierssen et al., 2009; Haydar and Reeves, 2011). We find that these cells fire action potentials (APs) in response to smaller current input and that the APs are narrower and have a higher overshoot. These differences may alter GC processing of signals conveyed to the cerebellum by MFs.

## 2 Results

### 2.1 Cerebellar GCs are larger in Ts65Dn mice

Whole-cell patch-clamp recording was used to determine if the electrical properties of mature cerebellar GCs (P40–60) are altered in the hypogranular cerebellum that characterizes DS. The data presented were obtained from slices derived from 10 Ts65Dn mice and 15 wild-type mice, which were littermates of the Ts65Dn mice. Measurements of input capacitance (*C*_in_) indicated that the surface area of the GCs recorded in this study was ~ 25% greater for cells from Ts65Dn DS mice than wild-type mice (median and inter-quartile values calculated from voltage deflections evoked by negative current jumps in current-clamp, wild-type, 3.0 (2.4, 4.0) pF, *n* = 48; Ts65Dn, 3.8 (3.1, 4.4) pF, *n* = 40, *p* = 0.008, Mann–Whitney *U* test; median and inter-quartile values of amplifier-readout after cancelation of current transients in voltage-clamp, wild-type, 2.1 (1.7, 3) pF, *n* = 48; Ts65Dn, 2.9 (2.5, 3.3) pF, *n* = 40, *p* = 0.033, Mann–Whitney *U* test). The increase in size of Ts65Dn GCs suggested by the difference in *C*_in_ is consistent with reports of a lower packing density of GCs in the Ts65Dn cerebellum (Baxter et al., 2000; Roper et al., 2006). As we did not anticipate a difference in *C*_in_, we did not examine cell morphology by filling cells with a dye during recording in order to determine if the increased *C*_in_ was due to enlargement of the soma or dendrites.

### 2.2 Increased excitability of Ts65Dn GCs

**Fig. 1:**
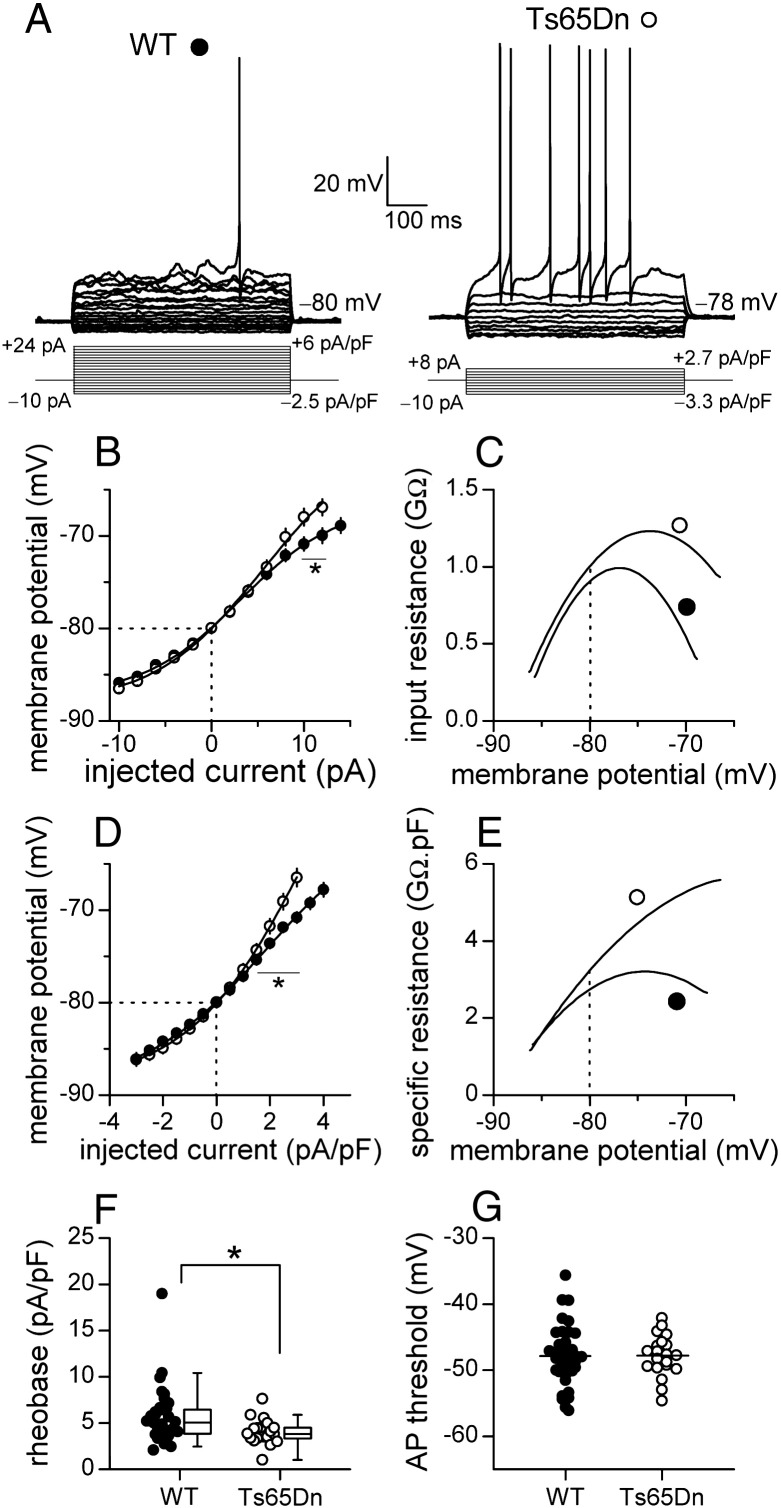
Intrinsic membrane properties of GCs are altered in Ts65Dn mice. A. Superimposed traces of changes in membrane potential of a mature wild-type (WT) cell (P50, filled circle) and a mature Ts65Dn cell (P53, empty circle) in response to constant current injections (once every 5 s, from − 10 pA in + 2 pA steps). Currents normalized by input capacitance are also given. B. Subthreshold voltage–current relationships for wild-type (*n* = 35–26) and Ts65Dn (*n* = 18–16) cells. The relationships differ at current injections above + 8 pA (*f_1_,_32_ = 4.21, *p* = 0.048, two-way repeated measures ANOVA). Solid lines are fitted sigmoidal curves. C. Plots of mean input resistance against membrane potential, obtained by differentiating sigmoidal curves in B. D. Relationships between subthreshold membrane potential and injected current-density. They differ above + 1 pA/pF (*f_1_,_41_ = 10.11, *p* = 0.003, two-way repeated measures ANOVA). Solid lines are fitted sigmoidal curves. E. Plots of mean capacitance-specific membrane resistance against membrane potential (first derivative of sigmoidal curves in D). F. Scatter plots and box plots comparing rheobase (the minimum current-density required to evoke APs) in Ts65Dn and wild-type GCs (median values: WT, 5.1 pA/pF, *n* = 37; Ts65Dn, 3.8 pA/pF, *n* = 20; *p = 0.007, Mann–Whitney *U* test). G. Scatter plots showing no difference in AP voltage-threshold (horizontal lines indicate mean values: WT, − 47.8 mV, *n* = 37; Ts65Dn, − 47.8 mV, *n* = 20; *p* = 0.972, Student's *t* test). Dashed lines in B–E indicate mean resting membrane potentials.

As described previously for wild-type cerebellar GCs (Brickley et al., 2001; Cathala et al., 2003; D'Angelo et al., 1995, 1998), current-clamp recording revealed a non-linear dependence of subthreshold membrane voltage on injected current in wild-type GCs (Figs. 1A and B). The relationship was also non-linear in Ts65Dn cells, but it was not identical to that in wild-type cells (Figs. 1A and B). While there was no difference in resting membrane potential (Fig. 1B, wild-type, − 80.0 ± 0.3 mV, *n* = 38; Ts65Dn, − 79.7 ± 0.5 mV, *n* = 21; *p* = 0.607, Student's *t*-test) or in voltage changes caused by hyperpolarizing currents, depolarizing currents caused greater voltage changes in Ts65Dn than in wild-type GCs (Fig. 1B). Hence, input resistance (*R*_in_) varied with membrane potential in both types of cells but *R*_in_ at depolarized membrane potentials was higher in Ts65Dn than in wild-type GCs. This divergence is more apparent when the mean *R*_in_, derived from the mean voltage–current relationship (Fig. 1B), is plotted against membrane potential (Fig. 1C).

If the higher *C*_in_ was the only difference between Ts65Dn and wild-type GCs, the *R*_in_ of Ts65Dn cells would be lower than that of wild-type cells at all membrane potentials. That this was not the case (Fig. 1C) indicates that the resistance of a unit area of membrane is higher in Ts65Dn GCs, and hence the density of open ion channels is lower. In order to compare membrane resistance, injected currents were normalized by *C*_in_, a measure of surface area, and expressed as current-density (pA/pF). Plots of subthreshold voltage against current-density were constructed (Fig. 1D), and the first derivative of the curve fitted to each of the mean voltage–current density relationships was plotted against membrane potential (Fig. 1E). These revealed the higher specific resistance in Ts65Dn GCs at voltages approaching the threshold for firing of APs (Fig. 1E), which resulted in a lower rheobase (size of the sustained current required to initiate AP firing, Fig. 1F). This was not accompanied by a difference in the voltage at which APs were triggered (Fig. 1G). These findings show that, once normalized for size, GCs fire more readily in Ts65Dn than in wild-type mice.

### 2.3 AP accommodation is unaltered in Ts65Dn GCs

**Fig. 2:**
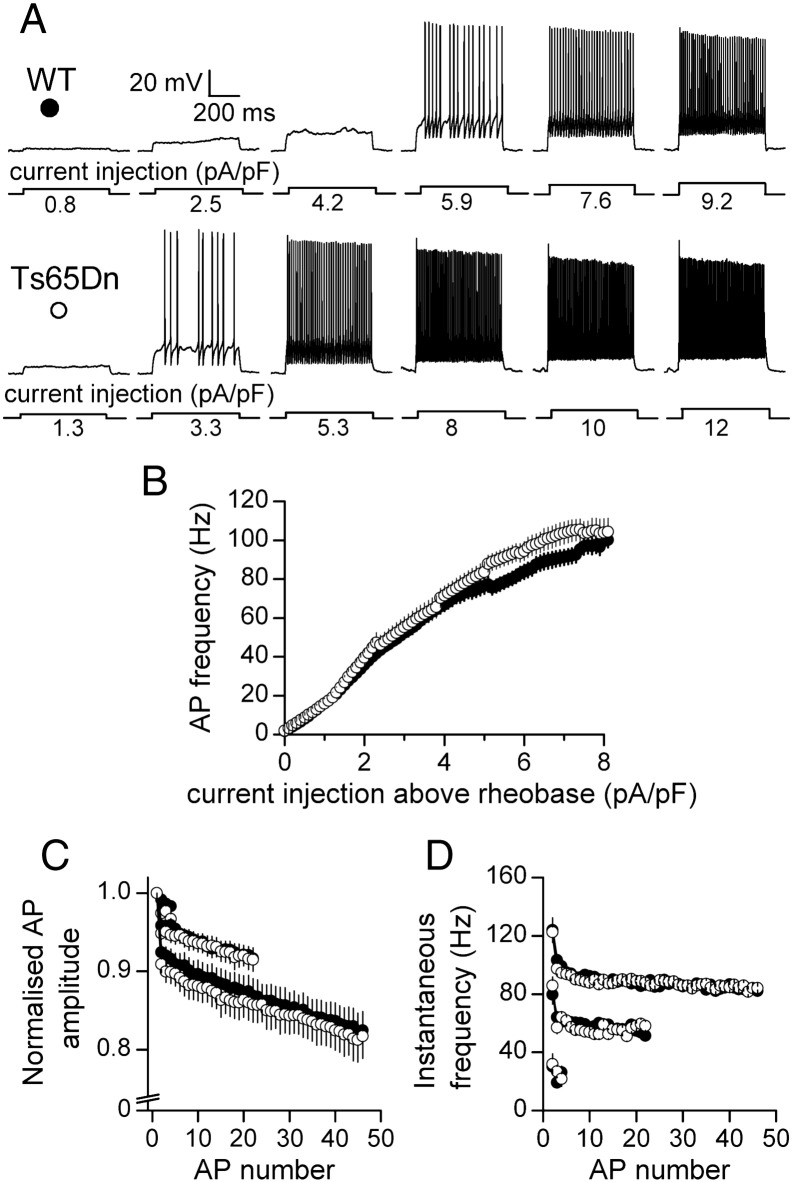
Enhanced excitability of GCs in Ts65Dn mice is not accompanied by a change in AP accommodation. A. Responses of a wild-type (WT, filled circle) and a Ts65Dn (empty circle) cell to increasing positive current injection, expressed as current-density. B. Dependence of AP frequency on magnitude of injected current-density above rheobase, in wild-type (*n* = 30–13) and Ts65Dn (*n* = 15–6) cells. C. Superimposed plots of AP amplitude relative to the first AP during current injections that evoked a minimum of 4, 22 and 46 APs (WT: *n* = 30, 30, and 25 cells; Ts65Dn: *n* = 20, 17 and 14 cells). D. Superimposed plots of instantaneous frequency for APs depicted in C.

Once depolarization exceeded AP threshold, increasing depolarizing current pulses increased the frequency of APs in both wild-type and Ts65Dn GCs (Fig. 2A). Equal increments in current-density caused a similar rise in firing frequency (Fig. 2B), indicating that a change in the steepness of the input/output relationship does not accompany the lower rheobase of Ts65Dn GCs outlined above. There was also no difference in AP accommodation, as deduced from comparisons of the attenuation of AP amplitude and instantaneous frequency during maintained depolarization. Fig. 2C shows heights of APs expressed as a fraction of the first AP for current injections that evoked a minimum of 4, 22 and 46 events. In both cell types, there was little change in the size of the 4 APs evoked near rheobase, but during suprathreshold depolarizations there was a marked decrease in amplitude between the first and second APs, which was followed by a gradual decline of subsequent APs, as observed previously in wild-type GCs (Brickley et al., 2001, 2007; D'Angelo et al., 1998; Hamann et al., 2002). Close superposition of the plots (Fig. 2C) demonstrates that attenuation of AP height during prolonged stimulation is not different in wild-type and Ts65Dn GCs. There was also no difference in firing pattern, as illustrated by close superposition of plots of instantaneous frequency against AP number (Fig. 2D). Furthermore, the first AP occurred with a similar latency at threshold at rheobase (wild-type, 182.9 ± 18.7 ms, *n* = 33; Ts65Dn, 181.9 ± 19.9 ms, *n* = 20; *p* = 0.973, Student's *t*-test) and the latency became shorter with increasing current injection in both wild-type and Ts65Dn GCs. At current injections double the strength of the rheobase (which were applied in a subset of cells), the mean latency to the first AP (the chronaxie) did not differ (median and interquartile values: wild-type, 9.5 (6.8, 9.5) ms, *n* = 29; Ts65Dn, 8.7 (6.9, 10.5) ms, *n* = 15; *p* = 0.310, Mann Whitney *U* test).

### 2.4 AP waveform is changed in Ts65Dn GCs

**Fig. 3:**
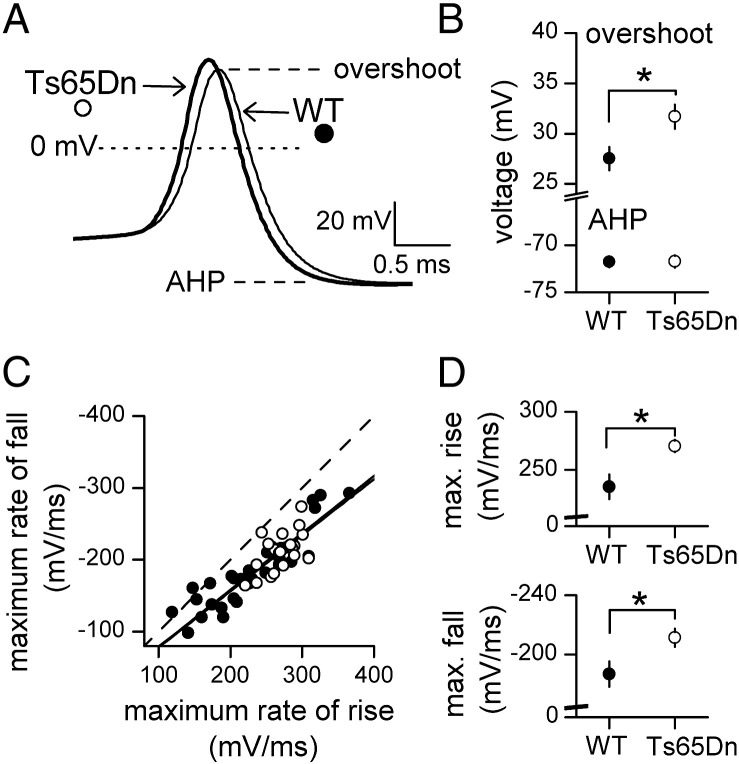
APs are larger and faster in GCs of Ts65Dn mice. A. Superimposed average APs from wild-type (WT, *n* = 33) and Ts65Dn (*n* = 20) cells, aligned on their threshold. B. Plot showing a higher mean overshoot in Ts65Dn cells (*p = 0.031, Student's *t* test) but no difference in mean afterhyperpolarization (AHP) (*p = 0.933, Student's *t* test). C. Plots of the maximum rate of fall against maximum rate of rise for APs evoked near the rheobase of individual cells. The slopes of the linear regressions (superimposed solid lines) for the two types of cells were less than − 1 (WT, − 0.79; Ts65Dn, − 0.78). The dashed line has a slope of − 1 and depicts the relationship if the rates of fall and rise during an AP are identical. D. Plots showing that the mean maximum rates of rise (upper) and fall (lower) are faster in Ts65Dn cells (rise: *p = 0.017, Student's *t* test; fall: *p = 0.048, Student's *t* test).

Although the increased excitability of Ts65Dn GCs was not accompanied by changes in AP accommodation, it was associated with changes in AP waveform (Fig. 3A). The average amplitude, measured between the overshoot and the afterhyperpolarization (Bean, 2007) for the first three APs evoked at or just above rheobase, was larger by 4.4 mV in Ts65Dn cells (wild-type, 99.4 ± 1.4 mV, *n* = 33; Ts65Dn, 103.8 ± 1.1 mV, *n* = 20; *p* = 0.032, Student's *t*-test). This was the result of a higher overshoot (by ~ 11%) without a change in afterhyperpolarization (Fig. 3B). The larger APs in Ts65Dn GCs were also ~ 10% narrower (width at half amplitude: wild-type, 714.9 ± 25.9 μs, *n* = 33; Ts65Dn, 643.5 ± 15.4 μs, *n* = 20; *p* = 0.045, Student's *t*-test). It has been shown previously that in wild-type GCs, membrane potential changes more slowly during the falling phase than the rising phase of the AP (Brickley et al., 2007). Fig. 3C shows that this difference was maintained in Ts65Dn cells, indicating that the speeding of the APs was due to a proportionate increase in the maximum rates of rise and fall, of ~ 13% (Fig. 3D). The finding that APs were faster in Ts65Dn cells, which have a longer membrane time constant because of their higher *C*_in_ and *R*_in_, indicates that the speeding reflects changes in ion channel activity or distribution, which overcomes the slowing effect of a longer membrane time constant on changes in membrane potential.

## 3 Discussion

It is known that there is a ~ 33% decrease in cerebellar volume and a 25–30% decrease in GC density in individuals with DS (Aylward et al., 1997; Baxter et al., 2000; Jernigan and Bellugi, 1990; Pinter et al., 2001; Raz et al., 1995). We have found that in GCs of young adult Ts65Dn mice (P40–60), which replicate cerebellar changes in DS (20% shrinking of cerebellar volume, 14% narrowing of the granular layer, 24% drop in GC density) (Baxter et al., 2000; Roper et al., 2006), the electrical properties of the surviving GCs are not identical to those of GCs in wild-type mice.

As the paucity of GCs in Ts65Dn mouse cerebellum and DS cerebellum stems from impaired division of precursor cells (Haydar and Reeves, 2011), changes in the electrical properties of Ts65Dn GCs could potentially be caused by arrested or slower development that results in immature electrophysiological characteristics. Wild-type GCs undergo marked changes in excitability, input resistance and AP waveform during postnatal development (Brickley et al., 2001; Cathala et al., 2003) but contrary to the notion that Ts65Dn GCs are an electrically immature version of wild-type GCs, Ts65Dn GCs have more negative resting potentials than immature wild-type GCs and they fire APs with faster rates of rise and fall and a larger amplitude, from a more hyperpolarized voltage-threshold (Brickley et al., 2001; Cathala et al., 2003; D'Angelo et al., 1995, 1998). In addition, the input resistance of Ts65Dn GCs changes with voltage, in contrast with the voltage-independent input resistance of immature wild-type GCs (Cathala et al., 2003).

Given that Ts65Dn mice are generated by triplication of a region of mouse chromosome 16 and are trisomic for genes orthologous to ∼ 104 of the ~ 310 genes present on human chromosome 21, which is triplicated in DS (Lana-Elola et al., 2011), changes in the electrical properties of Ts65Dn GCs could potentially be due to increased expression of ion channels encoded by trisomic genes. However, there is no obvious relationship between the voltage-dependent increase in input resistance or modified AP waveform and the ion channel-encoding genes present in three copies. Two of the trisomic genes are *Kcnj6* and *Kcnj15* which encode GIRK2/Kir3.2 and Kir4.2 potassium channels (Baxter et al., 2000), but GIRK2 protein expression is known not to be increased in cerebellar GCs of adult Ts65Dn mice (Harashima et al., 2006). By comparison, GIRK2 protein expression is increased in the hippocampus of adult and P14–21 Ts65Dn mice and this contributes to hyperpolarization of the resting potential (Best et al., 2011; Kleschevnikov et al., 2012). Furthermore, increased expression of GIRK2 or Kir4.2 channels due to gene dosage predicts decreased excitability and hyperpolarization of the resting membrane potential rather than the increased excitability and unchanged resting potential that we observed. A previous study reported that GIRK2 mRNA is elevated in cerebellar GCs of the TsCj1e mouse model of DS but this study was limited to young cells (P0–P10) and the functional impact of this upregulation was not examined (Laffaire et al., 2009). A third ion channel-coding trisomic gene is *Grik1* which encodes a kainate receptor subunit, but it is not clear how increased expression of this receptor in GCs would cause a voltage-dependent increase in input resistance or modify AP waveform.

Given the lack of trisomic genes in Ts65Dn mice that are known to encode ion channels, changes in the activity or expression of ion channels encoded by two-copy genes are likely to underpin the changes in AP waveform and excitability in Ts65Dn GCs. The higher overshoot, narrower width and faster rising and falling phases of APs are consistent with increased activity of voltage-gated sodium, potassium or calcium channels that generate AP in GCs (D'Angelo et al., 1998; Gabbiani et al., 1994; Saarinen et al., 2008). Previous studies have shown that in mature wild-type GCs, outwardly-rectifying TASK-3 potassium channels (*Kcnk9* on mouse chromosome 15) decrease input resistance more strongly at depolarized potentials, set the relatively negative resting potential, help maintain the overshoot and fast rising phase of APs, and sustain repetitive firing (Brickley et al., 2007). However, as we found no differences in resting potential and AP accommodation, and observed a speeding and augmentation rather than a slowing and reduction of APs in Ts65Dn GCs, it is unlikely that the voltage-dependent increase in input resistance in Ts65Dn GCs is explained by a decreased contribution of TASK-3 channels. The unchanged resting potential and unaffected firing frequency and pattern also exclude changes in other potassium channels (D'Angelo et al., 1998). Other studies have shown that the input resistance and excitability of mature wild-type GCs are also moderated by a tonic GABA_A_ receptor-mediated conductance (Brickley et al., 2001; Hamann et al., 2002) that does not alter resting membrane potential (Brickley et al., 2001). Our preliminary investigations (unpublished) suggest that a decrease in this tonic conductance may contribute to altered electrical properties of Ts65Dn GCs. This requires further investigation but if verified would be in contrast with the increased GABA-mediated phasic inhibition of CA1 pyramidal neurons in P14–21 Ts65Dn hippocampus (Best et al., 2011) and dentate granule neurons in adult Ts65Dn hippocampus (Kleschevnikov et al., 2012). However, the increased inhibition in CA1 neurons may be transient (Mitra et al., 2012) and inhibitory transmission in CA3 neurons of immature Ts65Dn hippocampus is reduced rather than enhanced (Hanson et al., 2007).

In contrast with our observations in adult Ts65Dn cerebellar GCs, AP shape in young (P14–21) Ts65Dn hippocampal CA1 neurons is unaltered (Best et al., 2011). However, APs and voltage-gated currents are modified in cultured dorsal root ganglion (DRG) neurons isolated from human DS (trisomy 21) fetuses, as well as in cultured DRG and hippocampal neurons from fetuses of Ts16 mice (a mouse model of DS which dies in utero). (Ts16 mice carry an extra copy of the whole of mouse chromosome 16 and are trisomic for a larger number of genes than Ts65Dn mice (Lana-Elola et al., 2011), but some of these trisomic genes are orthologous to genes on human chromosomes other than 21). The changes observed include faster and shorter APs in Ts16 mouse and trisomy 21 DRG cells (Ault et al., 1989; Caviedes et al., 1990) but slower and smaller APs in Ts16 mouse hippocampal neurons (Galdzicki et al., 1993), faster sodium currents with reduced inactivation in trisomy 21 DRG cells (Caviedes et al., 1990) but smaller sodium currents in Ts16 mouse hippocampal neurons (Galdzicki et al., 1993), and smaller and more slowly-activating calcium currents in Ts16 DRG cells (Caviedes et al., 2006) but increased calcium currents in Ts16 mouse hippocampal neurons (Galdzicki et al., 1998). Input resistance was usually unchanged but resting potential and input capacitance were affected in some studies but not in others (Ault et al., 1989; Best et al., 2011; Galdzicki et al., 1993, 1998). These and our findings in adult Ts65Dn cerebellar GCs support the idea that changes in neuronal function in DS can be executed by changes in the activity of ion channels encoded by two-copy genes, while the different modifications (no change, increase versus decrease, speeding versus slowing) observed in different types of neurons suggest that such modifications are cell-specific.

It is thought that one role of GCs is to filter the quantity of information conveyed to the cerebellum by MFs before passing it on to PCs and inhibitory interneurons (Arenz et al., 2009). This role is favored by a relatively low input resistance of the GCs, which dampens their excitability so that closely-timed inputs from one or more MFs are usually necessary to evoke GC firing (Cathala et al., 2003; D'Angelo et al., 1995; Hamann et al., 2002). Our finding that GCs in Ts65Dn mice are more excitable predicts weaker sparsification of MF signals (Hamann et al., 2002), as activation of fewer MF inputs would be needed to evoke GC firing. In addition, the increased amplitude and speeding of GC APs that we have observed may subtly modify the characteristics of glutamate release at downstream synapses between GC axons (parallel fibers) and PCs. These predictions need to be investigated experimentally, as changes in other properties, such as the probability of glutamate release from MFs and the amplitude and kinetics of excitatory postsynaptic currents (EPSCs), may mitigate the impact of enhanced GC excitability on MF–GC information transfer. A detailed study of synaptic transmission in the CA3 area of cultured or acute hippocampal slices of, respectively, P5 and P13–16 Ts65Dn mice revealed complex changes in excitatory and inhibitory synaptic transmission (Hanson et al., 2007). These included an increase in the number of excitatory synapses between CA3 pyramidal neurons and a decrease in the percentage of these synapses that was silent, a reduction in the amplitude of EPSCs at the active synapses, a diminished number of excitatory MF inputs and a reduction in inhibitory input from interneurons.

The impact of the changes in excitability and AP waveform that we have observed in Ts65Dn GCs on cerebellar function in humans with DS is unclear. If such changes accompany the decrease in GC number that occurs in all people with DS, they may result in altered GC signaling to downstream PCs that plays a part in the motor dysfunction displayed by most individuals with DS. Alternatively, such changes may compensate for the loss of GCs and minimize the degree of motor deficit that would otherwise occur. Different studies report either the presence (Costa et al., 1999; Turner et al., 2001) or absence (Baxter et al., 2000; Escorihuela et al., 1995; Hyde et al., 2001; Klein et al., 1996) of motor impairment inTs65Dn mice, making it difficult to ascribe roles for changes in GC number or electrophysiology to cerebellar dysfunction. It would be helpful to know if the electrical properties of cerebellar GCs are similarly altered in the Tc1 mouse model of DS, which carries much of human chromosome 21 and shows both impaired motor performance and a marked decrease in GC density (Galante et al., 2009; O'Doherty et al., 2005). Motor performance of the TsC1je mouse model of DS, which shows a smaller decrease in GC density and contains a smaller number of triplicated genes, has not been described (Moldrich et al., 2007). The cerebellum is also important for the production of fluent speech (Ackermann, 2008) and people with DS have difficulty in producing clear and ordered speech (Barnes et al., 2006) but this is one characteristic that cannot be assessed in mouse models of DS.

In addition to a reduced density of GCs in the Ts65Dn cerebellum, there is narrowing of the molecular layer, loss of PCs, and structural abnormalities in the axons of surviving PCs (Baxter et al., 2000; Necchi et al., 2008), but the electrical properties of these PCs have not been investigated. A previous study addressed the possibility that excitatory synaptic transmission on to PCs is altered inTc1 mice (Galante et al., 2009). It found no changes in the probability of transmitter release or EPSC waveform at synapses on PCs formed by afferent climbing fibers. It also found no changes in basal probability of glutamate release or in long-term depression of synaptic transmission at synapses between GC axons (parallel fibers) and PCs, although a slowing of EPSCs was reported. The slowing of the EPSC kinetics was not investigated in detail and the EPSC amplitudes were not compared, but it is consistent with the idea that changes in the properties of GCs, as we have observed, may alter signaling at downstream parallel fiber–PC synapses.

In summary, this study finds that the decrease in the number of cerebellar GCs in the Ts65Dn model of DS is accompanied by modification of the electrical properties of the GCs. Further studies are needed to determine if and how this affects processing of sensorimotor information by the cerebellum in DS.

## 4 Experimental procedures

### 4.1 Animals

Mice were generated by crossing female B6EiC3Sn a/A-Ts(17^16^)65Dn (Ts65Dn) mice, carrying a partial trisomy of chromosome 16 (Reeves et al., 1995), with C57BL/6JEi × C3H/HeSnJ (B6EiC3Sn) F1 males, at the University of Bristol. Parental generations of all three mice strains were obtained from The Jackson Laboratory (Bar Harbor, Maine, USA). To distinguish trisomic Ts65Dn from euploid littermate animals (wild-type), quantitative real-time polymerase chain reaction of tail-tip genomic DNA (Truett et al., 2000) was used to measure expression of the App gene (present in three copies in Ts65Dn and two copies in wild-type animals) relative to expression of the *Apob* gene (present in two copies in both Ts65Dn and wild-type animals; The Jackson Laboratory Protocols) (Liu et al., 2003).

### 4.2 Cerebellar slices

Parasagittal slices of cerebellar vermis (200 μm) were prepared from male Ts65Dn mice and wild-type mice (littermates of Ts65Dn mice) aged between postnatal day (P)40 and P60, on a Leica VT1000S vibrating microtome (Leica Microsystems, Nussloch, Germany). Slices were cut in ice-cold sucrose-based solution (in mM: 248 sucrose, 1.3 MgSO_4_, 5 KCl, 2.4 CaCl_2_, 1.2 KH_2_PO_4_, 26 NaHCO_3_, 10 d-glucose, pH 7.4, bubbled with 95% O_2_/5% CO_2_) and stored in standard Krebs–Henseleit solution (in mM: 124 NaCl, 1.3 MgSO_4_, 5 KCl, 2.4 CaCl_2_, 1.2 KH_2_PO_4_, 26 NaHCO_3_, 10 d-glucose, pH 7.4, bubbled with 95% O_2_/5% CO_2_) at room temperature prior to patch-clamp recording.

### 4.3 Patch-clamp recording and analysis

Current-clamp recordings were made with patch-pipettes (thick-walled borosilicate glass, coated with Sylgard 184, fire-polished) and an Axopatch 200B amplifier in fast current-clamp mode (Axon Instruments, Union City, CA), from slices superfused with Krebs–Henseleit solution at ~ 23 °C, in keeping with previous patch-clamp studies of granule cells at a similar temperature (Brickley et al., 2001, 2007; Cathala et al., 2003; Pugh and Jahr, 2011). Pipettes contained, in mM: 126 KCH_3_SO_3_, 4 KCl, 10 HEPES, 4 MgATP, 5 EGTA, 4 NaCl, 0.5 CaCl_2_, pH 7.2 with KOH, and had resistances of 4.5–8.5 MΩ. Constant current injections were applied once every 5 s, from − 10 pA in + 2 pA steps. Recordings of voltage were low-pass filtered at 10 kHz (4 pole Bessel filter on the amplifier), acquired at 62.5 kHz with a Cambridge Electronic Design (CED) power 1401 A/D interface and Signal software (CED, Cambridge, UK), and analyzed with Signal software and Origin software (Microcal, Northampton, MA). Membrane potentials were corrected for a calculated junction potential of 8.8 mV. Action potential (AP) parameters were measured for the first three APs elicited at or just above rheobase (the current injection required for initiation of APs) and averaged. Voltage-threshold and maximum rates of fall and rise were measured using phase-plane plots (supplementary Signal script, Steven Clifford, CED) (Bean, 2007). The first three APs evoked near rheobase were averaged for each cell, and these were averaged across cells to generate the ‘average wild-type AP’ and the ‘average Ts65Dn AP’.

The input capacitance (*C*_in_) of each cell was measured in two ways. One measure was calculated from the time-constant of a single exponential function fitted to the voltage deflection generated by a negative current injection (− 10 or − 8 pA) (D'Angelo et al., 1995). A second measure was taken from amplifier settings used to cancel current transients generated by 5 mV jumps in voltage-clamp mode, as in several previous patch-clamp studies of granule cells (Brickley et al., 2001; Cathala et al., 2003). GCs of all ages behave as a single electrical compartment and the measured *C*_in_ encompasses capacitances of the soma and dendrites (Cathala et al., 2003). The *C*_in_ calculated from fits to voltage-changes caused by negative current injections was used to express current as current-density (pA/pF). Equal increments in the size of the currents resulted in unequal increments in current-density in different cells, because of cell-to-cell variation in *C*_in_. Therefore, to enable averaging of plots of voltage or AP frequency against current-density, the plot for each cell was interpolated using equally-spaced points (0.5 or 0.1 pA/pF interval) and interpolated values were averaged.

The Shapiro–Wilk test was used to determine if data were normally distributed, before choosing a statistical test to compare differences using Origin or GraphPad Prism (La Jolla, CA) or SPSS (Chicago, IL). Differences were considered significant at *p* < 0.05. Data are summarized as mean ± standard error of the mean (SEM) or median and interquartile values (in parentheses), with *n* denoting number of cells. Symbols and error bars in figures represent mean ± SEM.
